# Metastasis Inhibition by Cell Type Specific Expression of *BRMS1* Gene under The Regulation of *miR200* Family Response Elements

**DOI:** 10.22074/cellj.2021.6988

**Published:** 2021-05-26

**Authors:** Samila Farokhimanesh, Mahdi Forouzandeh Moghadam, Marzieh Ebrahimi, Zahra Sadat Hashemi

**Affiliations:** 1. Department of Medical Biotechnology, Faculty of Medical Sciences, Tarbiat Modares University, Tehran, Iran; 2. Department of Biotechnology, Applied Biophotonics Research Center, Science and Research Branch, Islamic Azad University, Tehran, Iran; 3. Department of Stem Cells and Developmental Biology, Cell Science Research Center, Royan Institute for Stem Cell Biology and Technology, ACECR, Tehran, Iran; 4. Department of Medical Biotechnology, School of Advanced Technologies in Medicine, Tehran University of Medical Sciences, Tehran, Iran

**Keywords:** Breast Cancer, *BRMS1*, *MiR-200*, Neoplasm Metastasis

## Abstract

**Objective:**

Specific expression of therapeutic genes in cancer therapy has been per used for many years. One of the innovative strategies that have recently been introduced is employing miRNA response elements (MREs) of microRNAs (whose expression are reduced or inhibited in cancerous cells) into the 3´UTR of the therapeutic genes for their specific expression. Accordingly, MREs of anti-metastatic miRNA family have been used in 3´UTR of the metastasis suppressor gene in the corresponding cells to evaluate the level of metastatic behavior.

**Material and Methods:**

In this experimental study, 3´UTR of the *ZEB1* gene with 592 bp length, encompassing multiple MREs of *miR-141, miR-429, miR-200b* and *miR-200c*, was employed to replace *BRMS1 3´UTR*. The obtained vector was then assessed in the context of MCF-10A, MDA-MB231 and MCF-7 cells.

**Results:**

It was shown that the employed MREs are able to up-regulate *BRMS* expression in the metastatic MDA- MB231 cells (almost 3.5-fold increase), while it was significantly reduced within tumorigenic/non-metastatic MCF-7 cells. Specific expression of BRMS1 in metastatic cells led to a significant reduction in their migratory and invasive characteristics (about 65% and 55%, respectively). Two-tailed student’s t test was utilized for statistical analysis.

**Conclusion:**

It was demonstrated that a chimeric vector containing *BRMS1* which is regulated by miR-200 family response element may represent a promising therapeutic tool. This is due to the capability of the chimeric vector for cell type-specific expression of anti-metastatic genes with lowest side-effects. It consequently prohibits the invasive characteristics of metastatic cells.

## Introduction

Despite years of research, metastasis (a multi-steps process through which the primary tumor cells pervade neighbor tissues, while each of these steps requires tight regulation) is still considered as the cause of approximately 90% of the mortalities related to the cancer and for this reason, it has been particularly significant in the cancer treatment investigation. In this regard, up-regulation of the therapeutic genes in metastatic cancer cells have always been a major challenge (1).

Different strategies have been introduced for specific expression of therapeutic genes from which post-transcriptional targeting has attracted enormous interest. This targeting strategy can post-transcriptionally suppress gene expressions via establishing sequence specific interaction with the common miRNA response elements (MREs) over 3ˊ untranslated regions (3ˊUTRs) of the associated miRNA targets (2).

Discovery of the abnormal expression of miRNAs (down-regulation or up-regulation) in different steps of malignancy, among the various cancers, have been performed via the genome wide investigation methods, containing distinct micro-array platforms and bead-based flow-cytometry. This finding revealed that 3ˊUTR of the down-regulated miRNAs (which contain their microRNA target sequences) could be employed for specific expression (3, 4).

For targeting metastasis, miRNAs which are involved in epithelial-mesenchymal transitions (EMT) are thought to be the best choice, because EMT is one of the early steps to promote malignant tumor progression (5). The above procedure is defined by loss of epithelial features along with the achievement of mesenchymal characteristics. EMT could convert immotile epithelial cells into the motile mesenchymal types (6).

It should be noted that *miR-200* family has been recognized as one of the fundamental regulators of the epithelial phenotype by binding to zinc finger E-box binding homebox1 and 2 (*ZEB1* and ZEB2, respectively), two prominent transcriptional repressors of polarity (*CRB3* and *LGL2*) and cell adherence (*E-cadherin*) genes. Their expressions are significantly increased in metastatic cells which have mesenchymal characteristics. In the cells with epithelial characteristics, *miR-200* family members bind to their MREs on the *ZEB1* and *ZEB2* 3ˊUTR and inhibit their expressions. Using *ZEB1* 3ˊUTR (that include MREs of *miR-200* family), in the 3ˊUTR of a therapeutic gene as a post-transcriptional targeting moiety, would be an effective strategy. Using this strategy, specific expression of metastasis suppressor gene in the invasive cells could be occurred (7). This strategy has already been used for on colyticadeno-viruses to possess specific nature to glioma cell by *miR-128, miR-124, miR-218 and miR-146b* response elements, as well as for specific expression of* TRAIL* gene in uveal melanoma cells for growth suppression by *miR-34a, miR-137* and *miR-182 *response elements. The results have been quite satisfactory (8, 9).

In order to select a proper therapeutic gene, pleiotropic anti-metastatic genes are in priority. Due to its ability to regulate multiple steps of metastasis (pleiotropic anti-metastatic function), including metastatic colonization at the secondary tissue site which is believed to be a key vulnerability of metastatic cancer, the metastasis suppressor genes may be the most relevant choice for therapeutic intervention (10). One of the most applicable members of metastasis suppressor family, which has a great potential of metastasis inhibition, is the breast cancer metastasis suppressor 1 (*BRMS1*). 

*BRMS1* has been first described in 2000 following the observation that entering a typical, neomycin-tagged human chromosome 11 decreased metastatic potential of the MDA-MB435 human breast cancer cells by 70- 90% with no prevention of primary tumor growth (11). According to some studies, metastasis is repressed by *BRMS1* via inhibition of several stages throughout the process cascades such as migratory and invasive phenotype, colonization, angiogenesis, programmed cell death, cytoskeleton rearrangement, adhesion, gap junctional intercellular communication and increasing immune recognition by modulating numerous metastasis-related genes along with the metastasis-regulatory microRNA, called metastmiR. Some metastasis-related genes, which are regulated by *BRMS1* include: *urokinase-type plasminogen activator, fascin, epidermal growth factor receptor, osteopontin* and *C-X-C chemokine receptor 4* (12).

*BRMS1* also over-expresses *miR-146a, miR-146b *and *miR-335* which inhibit metastasis. It down-regulates *miR-10b, miR-373* and *miR-520c* which promote metastasis. It should be noted that some research found that metastasis suppressor genes have been previously employed for repressing metastasis of invasive cells and their results were promising (13, 14).

Therefore, re-expression of *BRMS1* affects both transcriptome and proteome (15-17). Considering these extensive roles, *BRMS1* could be a rational choice to pave the way for anti-metastatic therapy. In the present study, we exploited the differential profiles of miRNA expression among metastatic breast cancer cells and normal cells to confer specific *BRMS1* expression. Subsequently, we evaluated the possibility and efficiency of *miR-200* family response elements for regulating particular expression level of *BRMS1*. 

## Materials and Methods

### Cell culture

In this experimental study, three cell lines were obtained from ATCC (Manassas, USA) including non-tumuorigenic phenotype of MCF-10A, tumourigenic and non-metastatic phenotype of MCF-7 and metastatic phenotype of MDA-MB231 breast cancer cell lines. It should be noted that the medium selected for culturing MCF-10A cells is Dulbecco’s modified Eagle’s Medium (DMEM, Life Technologies Inc., USA)/F12 with 0.5 μg/ml hydrocortisone, 20 ng/ml epidermal growth factor (EGF), 100 ng/ml cholera toxin, 10 μg/ ml insulin and 5% donor horse serum as supplements. MCF-7 cell line was propagated in DMEM/F12, 1% penicillin/streptomycin (Gibco, USA) and 10% fetal bovine serum (FBS, Gibco, USA). MDA-MB231 cells have been grown in the conventional DMEM with 1% penicillin-streptomycin solution (Life Technologies Inc., USA) and 10% FBS as supplements in a moistened atmosphere of 5% CO_2_ .

### Extraction of RNA and quantitative reverse transcription polymerase chain reaction

Based on the pre-determined plan, total RNA was isolated from the three cell lines via the RNeasy mini kit (Qiagen, Germany). cDNA was primed in a randomized manner from total RNA through the RevertAid First Strand cDNA Synthesis Kit (Thermo Fisher Scientific, USA). Quantitative reverse transcription polymerase chain reaction (qRT-PCR) assay was implemented three times by SybrPremix Ex Taq II (Takara, Japan) on a Rotorgene 3000 series PCR device (Corbett Research, USA) using the following primers for *ZEB1* and *ZEB2*, in addition to the endogenous *BRMS1* gene:

*ZEB1*-

F: 5ˊ-GAG ATC AAA GAC ATG TGA CGC AG-3ˊ

R: 5ˊ-CTT CTC TCC ACT GTG AAT TCT TAA G-3ˊ

*ZEB2*-

F: 5ˊ-AGG GAC AGA TCA GCA CCA AAT G-3ˊ

R: 5ˊ-ACT CGT AAG GTT TTT CAC CAC TGT G-3ˊ

*BRMS1*-

F: 5ˊ-AGC TCT GAA TGG TGG GAT GAC-3ˊ

R: 5ˊ-CAC GAT GTA TGG GCC AGA AAC-3ˊ

After collecting the required information, Rotorgene software was used to analyze the data. Moreover, the comparative quantification feature of the Rotorgene software was used to determine the relative levels of expression. In addition, each mRNA quantification datum was normalized to *β-actin*. All fold changes in the expression were determined by using a comparative Ct (ΔΔCt) technique.

### Extraction of miRNA and quantitative reverse transcription polymerase chain reaction

Extraction of the total RNA, with effective recovery of small RNAs, was done in the three cell lines using miRCURY RNA isolation kit (Exiqon, Denmark). Then, cDNA was synthesized using the Universal cDNA Synthesis Kit (Exiqon, Denmark).

With regard to the company’s guideline, the mature form of *miR-200* family was detected using LNA microRNA Primer Sets and miRCURY LNA Universal RT microRNA PCR Kit (Exiqon, Denmark). In the next step, relative levels of expression were identified using the relative quantification feature of Rotorgene software. Then, U6 small nuclear RNA was employed as an internal control. Afterwards, comparative Ct (ΔΔCt) technique was applied for determining fold changes of expression. Finally, a melting curve was analyzed for all of the utilized primer collections, all of which exhibited a single peak. They represented specificity of the all experienced primers. All assessments were performed three times.

### Construction of plasmids

The 3ˊUTR sequence of the *ZEB1* was retrieved from UTRdb. According to the results of qRT-PCR for *miR-200* family and bioinformatics analysis, 592 bp (from nucleotide 756 to 1348) region in the central part of *ZEB1* 3ˊUTR sequence, containing four miRNA binding sites (*miR-141, miR-429, miR-200b* and *miR-200c*), was amplified by the following primers: 

5ˊ-CGACGCGTCGGATAAGGACAGCAAAATCATCAG-3ˊ

5ˊ-GACTAGTCAAAGTACATATGTCAGTAAGAAGGG-3ˊ

The PCR product was cloned into 3ˊUTR of luciferase in pmiR-REPORT Luciferase miRNA Expression Reporter (Ambion, USA) by MluI and SpeIrestriction enzymes (Roche Applied Science, Australia; miR-report. *ZEB1*). Control plasmid of pmiR-REPORT β-gal was employed to normalize the transfection. Fidelity of PCR cloning was confirmed by sequencing. The 592 bp fragment of *ZEB1* 3ˊUTR was also amplified using the following primers:

5ˊ-CGCGTCGACGATAAGGACAGCAAAATCATCAG-3ˊ

5ˊ-CGGGATCCAAAGTACATATGTCAGTAAGAAGGG-3ˊ

Product of the amplification was cloned into 3ˊUTR of GFP in the control plasmid of pcDNA 6.2-GW/EmGFPmiR-neg (pc, Invitrogen, USA) through BamHI and SalI restriction enzymes (pc.Z, Roche Applied Science). Verification of PCR cloning was performed by sequencing.

It should be noted that optimization of *BRMS1* gene sequence was performed by GenScript (Genscript Corporation Piscataway, USA) in order to reach the highest probable level of expression. Afterwards, the optimized gene was cloned into both pc and pc.Z plasmids using the restriction enzymes SalI and DraI, which were called pc.BR and pc.BR.Z respectively. The accuracy of cloning was confirmed by sequencing.

### Luciferase reporter assay

5×10^4^ MCF-7 and MDA-MB231cells were plated in 24- well plates. Then, they were incubated overnight. Both cell lines were co-transfected in a 24-well plates with 0.10 µg of the pmiR-report. *ZEB1* luciferase reporter vector and 0.05 µg of the normalization plasmid pmiR-REPORT β-gal using the Lipofectamine 2000 (Invitrogen, USA). Lysis buffer was used to process the cells. Afterwards, luciferase activities were measured using Dual-Glo Luciferase Assay System (Promega, USA), 24 hours post-transfection. GFP reporter assay was also performed using standard protocol. It should be mentioned that the luciferase activities are presented as the average of three independent tests.

### miRNA mimics and inhibitors

*miR-200b*, *miR-200c, miR-141 *and *miR-429* mirVana™ mimics and inhibitors (Invitrogen, USA) were completely mixed and added to the cells (5×10^4^ MDA-MB231 and MCF-7 cells) with concentration of 40 nM (10 nM for each mimic or inhibitor) using the Lipofectamine™ 2000 based on the company’s guidelines. Twenty four hours later, the cells were transfected with pc, pc.BR and pc.BR.Z. Then, expression of *BRMS1* was assessed in these cells by qRT-PCR following the optimized specific primers (exogenous) for *BRMS1* genes:

5ˊ-TACGAACGGAGAAGGAGCGA-3ˊ

5ˊ-CGCTCTGCTCCGACTTCCTCC-3ˊ

All experiments were repeated three times.

### Transfections

The 24-well plates were used to plate 5×10^4^ cells of all three cell lines. Then, they were incubated overnight. MDA-MB231 and MCF-7 cells were transiently transfected by pc, pc.Z, pc.BR and pc.BR.Z, using lipofectamin 2000 for the subsequent experiments. Each transfection was carried out three times.

### Trans well migration assay

In order to assess migration, 2.5×10^4^ cells of three cell lines, which were transfected by four constructs (pc, pc.Z, pc.BR and pc.BR.Z) and serum starved cells, were plated into the upper chamber on the non-coated membrane (24- well insert, pore size 8 μm, Millipore Billerica, USA). Then they were allowed to migrate toward medium which contains serum in the lower chamber. When they were incubated at 37˚Cin a 5% CO_2_ humidified incubator for 24 hours, the cells on top of the chambers were eliminated via wiping with a cotton swab. Then, the migrated cells to the lower surface of filter were fixed in 4% formaldehyde for 30 minutes. Afterwards, 0.5% crystal violet was used to stain for 10 minutes. Next, cell migration was scored by counting 10 random fields per filter below a light microscope at ×40 magnification. Each assay was repeated three times.

### Trans well invasion assay

Matrigel-coated Trans well cell culture chambers (8 μm pore size) were used to analyze cell invasion. Concisely, transfected cells (2.5×10^4^ cells/well) were serum starved for 24 hours. Then, they were plated on the top of insert of a 24-well chamber in a medium without serum. Afterwards, the medium with 10% serum was added to the lower wells. Next, incubation of the cells was done for 24 hours. The cells on the upper side of filters were then mechanically removed by scrubbing with a cotton swab. As the last step, 4% formaldehyde was used to fix the membrane for 30 minutes and 0.5% crystal violet was utilized for 10 minutes. Ultimately, counting the invasive cells were performed at ×40 magnification from 10 different fields of each filter. Invasion assays were done in triplicate.

### Western blotting

pc.BR construct was used to transfect the MDA-MB231 cells. After 48 hours, the cells were lysed in radio-immunoprecipitation assay (RIPA) buffer (50 mM Tris-HCl pH=7.4, 150 mM NaCl, 1 mM EDTA, 0.1% SDS, 1% sodium deoxycholate and 1% NP-40). The buffer was enriched with cocktail of protease inhibitors (PMSF). Then, a cell scraper was used to scrape the cells. Afterwards, the cells were transferred into the ice cold tube for a brief sonication. Total protein was obtained by centrifuging the extract at 14,000 g at 4˚Cfor 10 minutes. MILLIPORE ultrafiltration column was used to obtain higher concentrations of the protein. It should be noted that Bicinchoninic acid assay (Thermo Fisher Scientific, USA) was used to measure concentration of the protein. The protein sample (40 µg) was isolated on a 12.5% SDS-polyacrylamide gel and transferred electro-phoretically onto Nitrocellulose Transfer membranes (PROTRAN, Schleicher & SchuellBioScience, Germany). Then, 3% skimmed milk in Tris-buffered saline/0.05% Tween-20 was used to block the membrane for one hour. Next, rabbit horseradish peroxidase-conjugated anti-BRMS1antibody (isotype: Ig G, Abcam, UK) was used to blot it for one hour. Ultimately, the augmented chemiluminescence detection kit (Thermo Fisher Scientific, USA) was employed to visualize the protein bands. Western blotting was repeated in triplicate.

### Statistical analysis

In order to statistical analyses of the present data, the two-tailed student’s t test was utilized. An asterisk means significant that shows P<0.05. Prism 6 statistical software (GraphPad Software, Inc.) was used for all graphs and statistical analyses. The results are expressed as mean ± standard deviation. Each experiment was repeated three times. 

### Ethical considerations

The study does not contain any experimental animals or human participants. It should be noted that each procedure has been implemented based on the Ethical guidelines of Faculty of Medical Sciences, Tarbiat Modares University, Tehran, Iran (code: 52112234).

## Results

### Differential ZEB factors and *miR-200* family expression profiles between metastatic and normal breast cells

**Fig.1:**
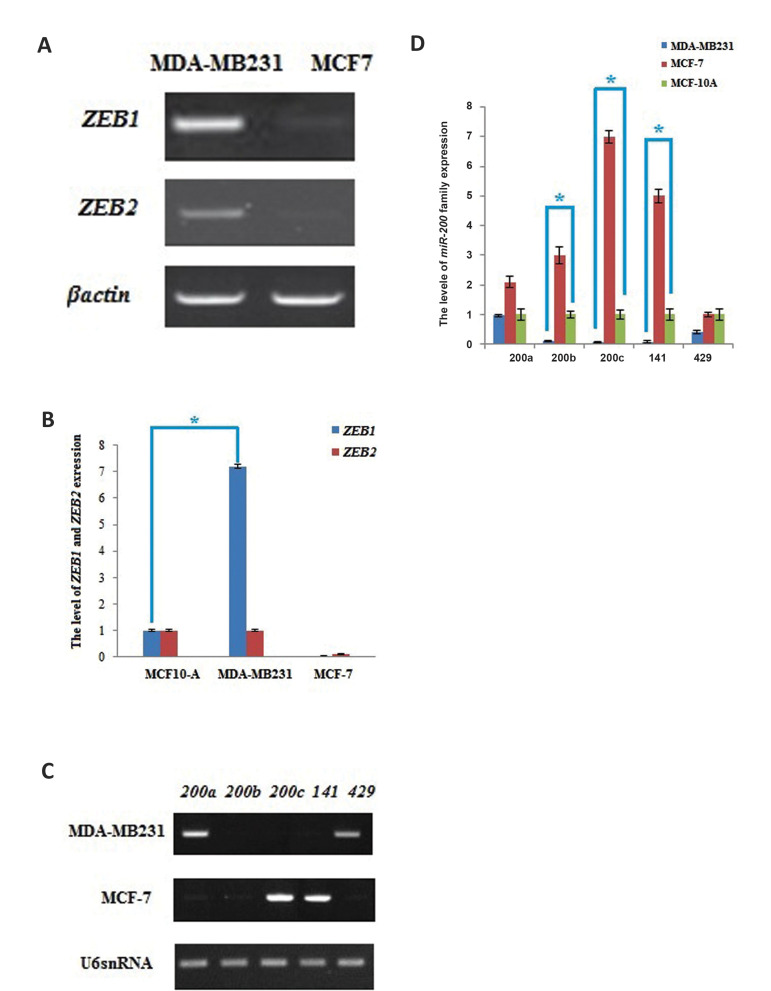
Differential ZEB factors and miR-200 family expression profiles between metastatic and normal breast cells. **A. ***ZEB1* and *ZEB2* mRNA detections using qRT-PCR method in untreated MDA-MB231 and MCF-7. **B. ***ZEB1* and *ZEB2* expression levels in MDA-MB231 (cancerous, metastatic cell line) and MCF-7 (cancerous, non-metastatic control cell line) relative to MCF-10A (normal cell line). **C. **qRT-PCR of *miR-200* family in MDA-MB231 and MCF-7 cells. **D.** The level of *miR-200* family expression in MDA-MB231 and MCF-7 relative to MCF-10A. Data represent means ± SD of three separate tests. *; P value for each condition was significant in comparison with the normal cells. qRT-PCR; Quantitative reverse transcription polymerase chain reaction.

Since *ZEB* 3ˊUTRs have the *miR-200* family-response elements, their expression profiles were investigated in MDA-MB231 and MCF-7 cells by qRT-PCR assays. The outputs of qRT-PCR assays showed that level of *ZEB1* expression was 7.2 fold higher than *ZEB2* in the metastatic cells compared to the non-metastatic cells (Fig .1A, B). Since the 3ˊUTR of the *ZEB* gene with higher expression level, is a better choice (due to the less inhibition by *miR-200* family), 3ˊUTR of *ZEB1* gene was selected. Then, expression profiles of *miR-200a, miR-200b, miR-200c, miR-141* and *miR-429* were investigated by qRT-PCR in the MDA-MB231 and MCF-7 cells relative to the non-tumorigenic MCF-10A. It was demonstrated that the levels of four out of five miRNAs (*miR-200b, 200c, miR-141* and *miR-429*) were significantly reduced in the tested metastatic MDA-MB231 cells compared to the cancerous but non-metastatic MCF-7 cells. This was consistent to the previously published data (Fig .1C, D, P<0.05) (18). The reduced expression levels of four microRNAs possibly ensure that using their MREs result in expressing the intended exogenous genes in metastatic breast cancer cells instead of non-metastatic and normal cells.

### Application of *miR-200b, miR-200c, miR-141 and miR-429* MREs confined exogenous gene expression within the metastatic cancer cells

**Fig.2:**
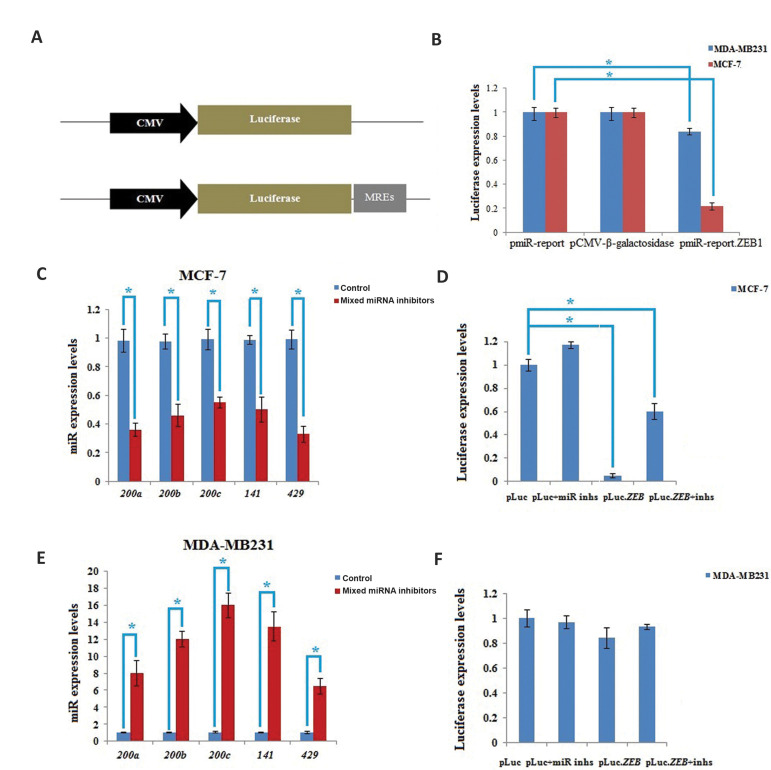
Use of MREs of miR-200 family confined exogenous gene expression within the metastatic cancer cells. **A.** Illustration of the structure of luciferase reporter plasmids. **B. **Evaluation of luciferase expression in MDA-MB231 and MCF-7 cells after the transfection of pmiR-REPORT β-gal control plasmid and pmiR-report ZEB1. **C. **Synthetic inhibitors of *miR-200b, miR-200c, miR-141 *and *miR-429 *were mixed and transfected into non-metastatic MCF-7. Expression levels of these miRNAs were assessed by qRT-PCR with U6, as endogenous reference and they were shown as values relative to the control groups. **D.** Co-transfection of MCF-7 cells with the indicated constructs and mixed miRNA inhibitors or controls. Twenty four hours later, luciferase expression was evaluated. Relative luciferase activity in the cells transfected with pmiR-report ZEB and control inhibitors was considered as standard. **E.** Synthetic mimics of *miR-200b, miR-200c, miR-141* and *miR-429* were mixed and transfected into MDA-MB231. Expression levels of these miRNAs were assessed by qRT-PCR with U6, as the endogenous reference and they were shown as values relative to the control groups. **F. **Co-transfection of MDA-MB231 with the indicated constructs and mixed miRNA mimics or controls. Twenty four hours later, luciferase expression was evaluated. Relative luciferase activity in the cells transfected with pmiR-report ZEB and control inhibitors were considered as standard. Data represent means ± SD of three separate tests. *; P<0.05 and qRT-PCR; Quantitative reverse transcription polymerase chain reaction.

For assessing whether MREs could be used for the specific expression of exogenous gene (*BRMS1*) in metastatic breast cancer cells, a reporter plasmid including luciferase regulated by their MREs was successfully constructed (Fig .2A). Results demonstrated that luciferase activity was not significantly changed in the pmiR-report. *ZEB1* transfected MDA-MB231 cells. However, its activity was markedly repressed in the MCF-7 cell line (Fig .2B). To confirm control of *miR-200b, miR-200c, miR-141* and* miR-429* on the exogenous gene expression under their respective MREs, assaying the luciferase was done in the pmiR-report. *ZEB1*-transfected cells after changing level of the above miRNAs. Expressions of endogenous *miR-200b, miR-200c, miR-141* and* miR-429 *were inhibited by 30-50% in MCF-7 through mixing the above four microRNA inhibitors. Thus, expression of luciferase was considerably up-regulated in pmiR-report. *ZEB1*-transfected cells (Fig .2C, D). Consistently, luciferase expression was almost 50% declined in pmiR-report. *ZEB1*-transfected MDA-MB231 cells, where by *miR-200b, miR-200c, miR-141* and *miR-429* levels were increased by treating with the mixture of four microRNA mimics (Fig .2E, F). These outputs showed that MCF-7 cells had higher endogenous expression of *miR-200* family than MDA-MB231 cells. So, using four microRNA inhibitors could inhibit them and luciferase activity was increased. However, in MDA-MB231 endogenous expressions of *miR-200* family were very low, using four microRNA mimics, which could bind to the MREs. This caused reduction of luciferase expression (Fig .2E, F, P<0.05).

### MREs of *miR-200b, miR-200c, miR-141 *and *miR-429* ensured expression of *BRMS1* specifically in MDA-MB231 cells

**Fig.3:**
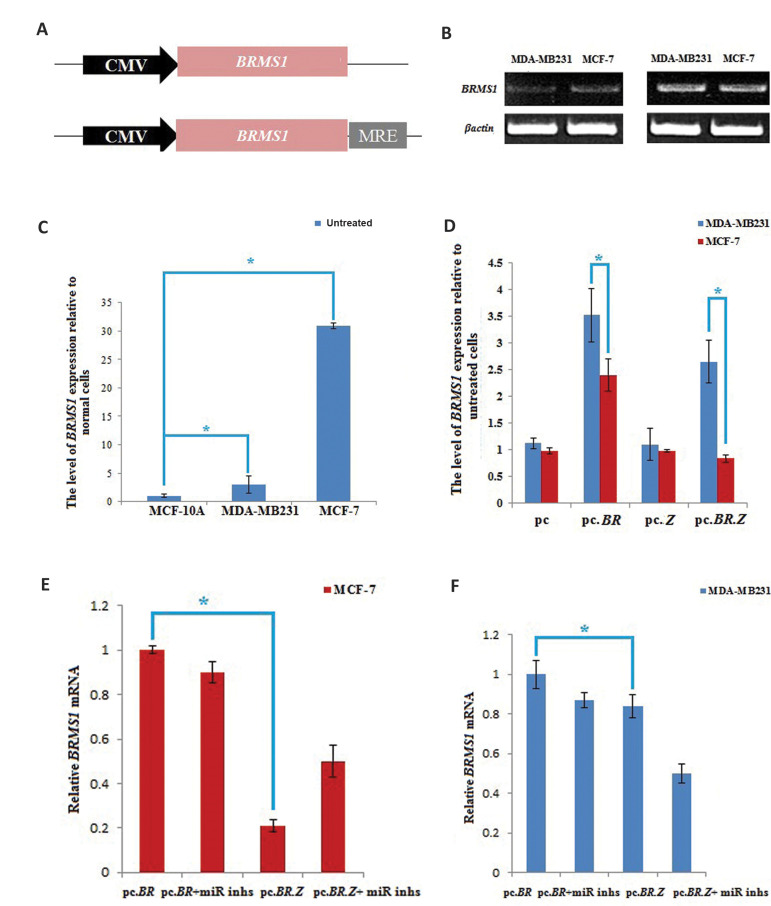
MREs of miR-200 family guaranteed particular expression of BRMS1 in MDA-MB231 cells and Pc.BR.Z mediated *BRMS1* expression depends on the quantity of miR-200 family. **A.** Illustration of the structure of chimeric vectors containing *BRMS1*.** B.** Semi-quantitative RT-PCR of *BRMS1*. *BRMS1* expression level was evaluated in untreated MDA-MB231 and MCF-7 cells (the endogenous level of *BRMS1*) and after transfection (ectopic level of *BRMS1*). **C. **qRT-PCR assay in untreated MDA-MB231 and MCF-7 cells and *BRMS1* expression level in untreated MDA-MB231 and MCF-7 relative to the normal cells. Data represent means ± SD of three separate tests (*; P<0.05). **D. ***BRMS1* mRNA expression level analysis using qRT-PCR assay in MDA-MB231 and MCF-7 cells transfected with pc, pc.Br, pc.Z and pc.Br.Z. **E.** MCF-7 cells were transfected with pc.Br and pc.Br.Z as well as the mixed inhibitors of miR-200 family. After 24 hours, expression level of *BRMS1* was assessed using qRT-PCR assay. **F.** MDA-MB231 cells were transfected with pc.Br and pc.Br.Z as well as the mixed mimics of miR-200 family. After 24 hours, expression level of *BRMS1* was assessed using qRT-PCR assay. *β-actin* was used as endogenous reference. Data represent means ± SD of three separate tests. P value for each condition was significant, compared to the untreated cells.

MREs were subsequently inserted into *BRMS1*expressing pc vector to regulate expression of the aforementioned metastasis suppressor gene. A chimeric plasmid was constructed by inserting 592 bp of *ZEB1* 3ˊUTR containing MREs of *miR-200b, miR-200c, miR-141 *and *miR-429*, immediately following the *BRMS1* open reading frame coding region (Fig .3A). Expression level of *BRMS1* was assessed in MCF-7 and MDA-MB231 before and after treatment by pc.BR semi-quantitative RT-PCR and qRT-PCR assays. Findings revealed that expression level of *BRMS1* in untreated MCF-7 cells was 10 fold more than MDA-MB231 cells. The results also confirmed increase of in *BRMS1* expression level (more than 3 fold) after transfection by pc.BR construct (Fig .3B, C). qRT-PCR assay showed that chimeric construct of the pc.BR.Z had almost the same levels of *BRMS1* gene expression as pc.BR in MDA-MB231, where as it was considerably inhibited (more than 2 fold decrease of *BRMS1* expression) in pc.BR.Z transfected MCF-7 cells (Fig .3D). These results were compatible to ourexpectation, since MDA-MB231 cells did not have *miR-200* family. So, when they were treated with pc.BR.Z, there was almost no *miR-200* family for binding to *ZEB1* 3ˊUTR and it could inhibit *BRMS1* expression. However, due to the*miR-200* family expression, expression of *BRMS1* was inhibited in MCF-7 (Fig .3D, P<0.05).

### Pc.BR.Z mediated *BRMS1* expression depends on the abundance of miRNA-200b, *miR-200c, miR-141* and *miR-429*

To test if the *BRMS1* expression by pc.BR.Z was depend on the levels of *miR-200b, miR-200c, miR-141 *and *miR-429*, synthetic miRNA inhibitors and mimics were added to the MDA-MB231 and MCF-7 cells. Then, *BRMS1* expression was evaluated in these cells using qRT-PCR assays. In MCF-7, which has higher levels of four microRNAs expression, *BRMS1* expression was significantly inhibited, after transfecting the cells with pc.BR.Z. Nonetheless, treating the pc.BR.Z transfected MCF-7 cells with microRNA inhibitors resulted in partially restoring *BRMS1* expressions (almost more than 2 fold increase in *BRMS1* expression). This increase is owing to the reason that microRNA inhibitors could bind to *miR-200* family and prevent them from attaching to their MREs, so *BRMS1* expression could be performed (Fig .3E). Consistently; transfecting MDA-MB231 cells with microRNA mimics remarkably decreased expression of *BRMS1* (almost 2 fold) in these cells, where by the endogenous levels of *miR-200b, miR-200c, miR-141* and *miR-429 *were low. But, microRNA mimic could bind to MREs and inhibit expression of *BRMS1*. Collectively, pc.BR.Z mediated *BRMS1* expression by the abundance of *miR-200b, miR-200c, miR-141* and *miR-429* (Fig .3F, P<0.05). 

### pc.BR.Z reduced migration and invasion of the metastatic breast cancers cells without affecting normal cells

**Fig.4:**
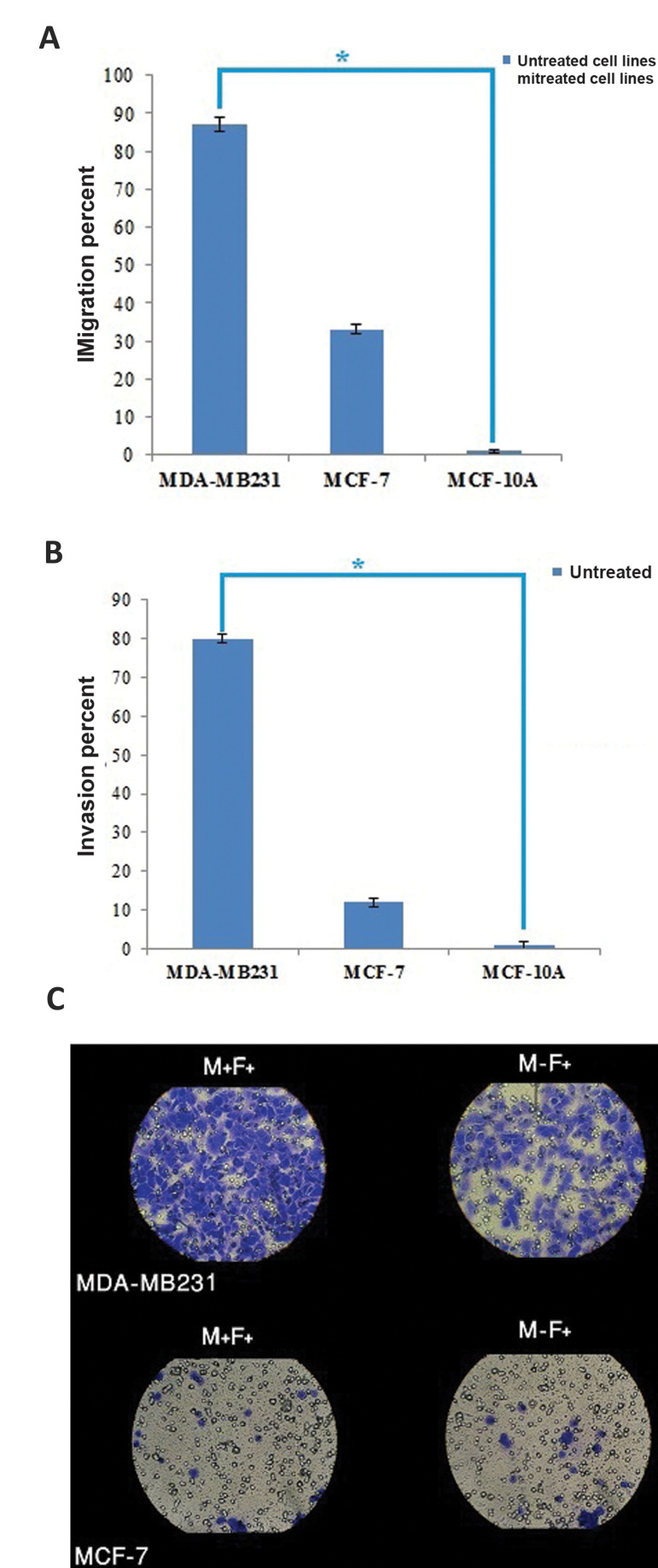
Migration and invasion assays before any treatment. **A.** Migration percent of MDA-MB231 and MCF-7 cells before any treatment. **B.** Invasion percent of MDA-MB231 and MCF-7 cells before any treatment. As it is shown, level of migration and invasion in MDA-MB231 cells are significantly more than MCF-7 without any treatment. C. Trans well migration assay and matrigel invasion assay in MDA-MB231 and MCF-7 cells before any treatment. Data represent means ± SD of three separate tests. *; P<0.05, M+F+; Contain matrigel and FBS, M-F+; Without matrigel and contain FBS. One out of 10 field as a sample (M-F+ indicates the level of migration and M+F+ indicates the level of invasion).

**Fig.5:**
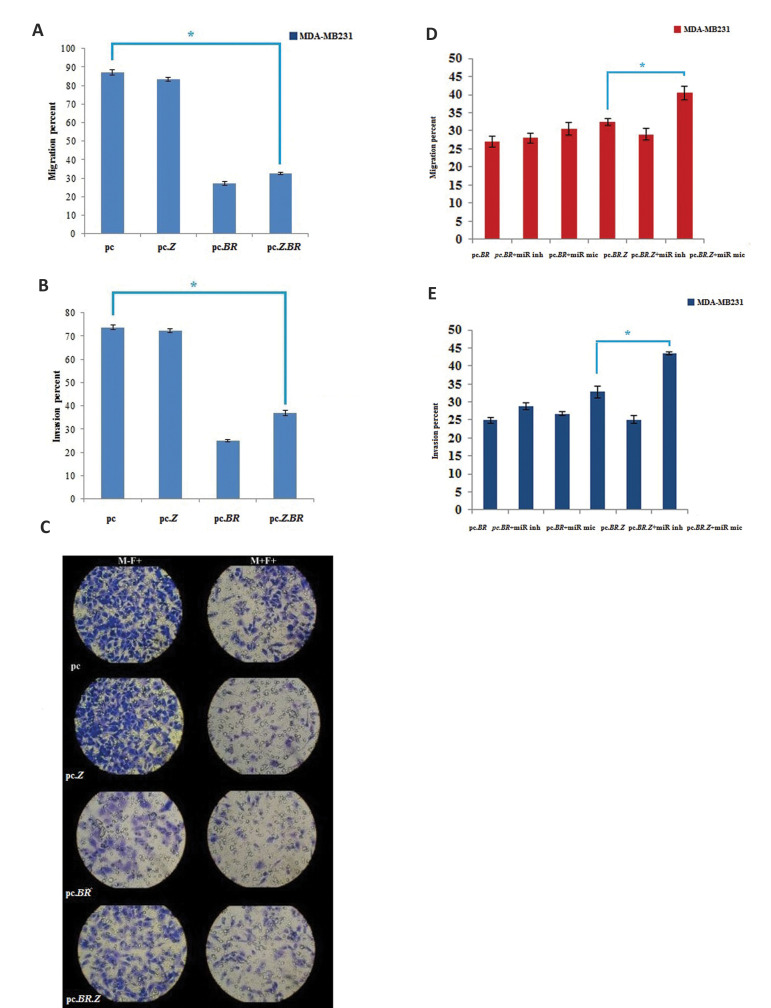
Migration and invasion assays after transfections. A. Migration percent after transfection of MDA-MB231 cells by four constructs. B. Invasion percent after transfection of MDA-MB231 cells by four constructs. C. Matrigel invasion assays in MDA-MB231 cells after transfection by four constructs. M+F+; Containing matrigel and FBS, M-F+; Without matrigel and containing FBS. One out of 10 field as a sample. D. Migration percent in MDA-MB231 cells transfected with pc.BR, pc.BR+ miR inhibitor, pc.BR+ miR mimic, pc.BR.Z, pc.BR.Z+ miR inhibitors and pc.BR.Z+ miR mimic. E. Invasion percent in MDA-MB231 cells transfected with pc.BR, pc.BR+ miR inhibitor, pc.BR+ miR mimic, pc.BR.Z, pc.BR.Z+ miR inhibitors and pc.BR.Z+ miR mimic. Data represent means ± SD of three separate tests. *; P<0.05.

To examine whether pc.BR.Z could decrease migration and invasion of metastatic breast cancer cells, we performed *in vitro* analysis specifically expressing *BRMS1* metastasis suppressor gene in the context of a chimeric pc.BR.Z vector in the MCF-7 and MDA-MB231 cells. qRT-PCR analysis demonstrated that *BRMS1* was increased (3.5 fold) in the metastatic cells transfected with pc.BR.Z, compared to the non-metastatic cells (Fig .3D). Then, assaying trans well migration and invasion were done on the untreated cells (Fig .4). The results indicated that migration rate in MDA-MB231 was 2.6 fold more than MCF-7cells (Fig .4A) and the invasion rate was 6.7 fold more than MCF-7 in the non-transfected cells (Fig .4B, C). Subsequently, we tested whether *BRMS1* had effects on the migration and invasion of MDA-MB231 cells, transfected with pc, pc.Z, pc.BR, pc.BR.Z or non-transfected cells. Pc.BR decreased the rate of MDA-MB231 cells migration and invasion of by 68 and 62.3%, respectively. pc.BR.Z also reduced these rates by 65 and 55%, respectively compared to pc and pc.Z transfected cells (Fig .5A-C). Levels of migration and invasion were decreased in the treated cells with pc.BR.Z. This may be due to the little leakage of *miR-429* expression. We also checked migration and invasion rates in MDA-MB231 cells transfected with pc.BR, pc.BR.Z, mixed mimics and inhibitors. It was demonstrated that there is almost more than 10% difference in migration and invasion of pc.BR.Z and pc.BR.Z+mimics, because miR-mimic could bind to MREs and inhibit the expression of *BRMS1*. This caused an increase in migration and invasion of the treated cells. Since the migration and invasion rates of untreated MCF-7 cells were negligible, their transfection with the constructs seemed to be futile (Fig .5D, E, P<0.05).

### Protein expression level

**Fig.6:**
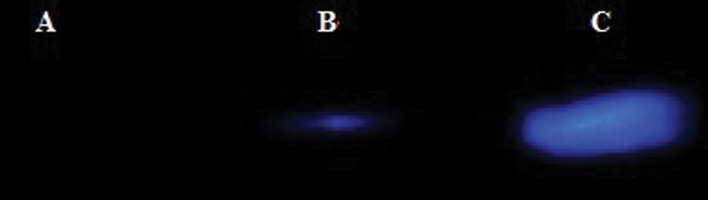
Chemiluminescent western blotting for protein expression levels. A. is the MDA-MB231 cell lysis without any *BRMS1* antibody (horseradish peroxidase-conjugated antibody (Abcam Company) treatment as a negative control group. B. Is the MDA-MB231 cell lysis with the *BRMS1* antibody treatment. and C. Is the MDA-MB231 cell lysis which was transfected by pc.BR construct, with the *BRMS1* antibody treatment.

BRMS1 protein level, encoded by pc.BR construct, was evaluated using western blot method after transfection. Figure 6 shows the western blot result for the total protein sample extracted from pc.BR transfected cell. These results indicated successful expression of the BRMS1 at the protein level (Fig .6, P<0.05).

## Discussion

Contemporary, MRE regulated approaches have garnered a lot of attention as an alternative gene therapy strategy for specific targeting of the malignant cells. MREs are more advantageous over the conventional gene therapy approaches (like transcriptional targeting approach or using cancer-specific promoters), offering higher efficacy and specificity for the certain cell types. Specific anti-metastatic microRNAs have been exhibited to be down-regulated in metastatic breast cancer cells (19, 20). Therefore, MREs corresponding to the aforementioned microRNAs might be applied to drive specific expression of well-established anti-metastatic genes in cancer cells and ultimately inhibit their invasiveness. Given these circumstances, we have devised a MRE regulated gene therapy strategy to inhibit invasiveness behavior of metastatic breast cell lines by specific expression of *BRMS1* gene. It has been demonstrated that a MREs-regulated vector containing *BRMS1* gene could be a compelling tool attaining this purpose.

*BRMS1* is among the promising anti-metastatic breast cancer genes which selectively suppresses metastasis without suppression of any cancer cell tumorigenicity. Pleiotropically acting *BRMS1* prevents multiple steps of the metastatic cascade. Diversity of *BRMS1* actions, employing a variety of mechanisms, contribute to its robust inhibition of metastasis. The recent reports have shown that *BRMS1* remarkably suppressed migration and invasion of cells in many types of cancer. Analysis of tissue micro-array of the patients revealed that *BRMS1* was considerably down-regulated in glioma cells in comparison with the normal astrocytes. Additionally *BRMS1* over-expression could inhibit migration and invasion of glioma cells via suppressing MMP-2 , NF-κB and uPA (21). In the other work, it was demonstrated that up-regulation of *BRMS1* decreased SDF-induced migration by reducing NF-κB dependent CXCR4 expression in NSCLC cell line (22). Rectal cancer xenograft invasiveness could also be reduced by over-expression of BRMS-1 (23). Besides, investigations on breast cancer showed that there is a reverse association between *BRMS1* over-expression and disease progression. Down-regulation of fascin, which is an actin-bundling protein, by *BRMS1* has been shown in another study. This exerted an inhibitory effect on metastasis of ovarian cancer cells (24, 25). All of the previously found data were in accordance with the present work in terms of reducing level of migration and invasion by up-regulating BRMS-1.

It confers activity of BRMS1 via regulating numerous metastasis-associated genes and microRNAs chiefly due to the altered SIN3: histone deacetylase chromatin remodeling complexes (26). Since *BRMS1* expression could induce various alterations at the molecular (transcriptome and proteome) levels and it is capable of inducing different phenotypic alterations like changing cyto-architecture (cell topography and ultrastructure), up-regulation of that may have undesirable effects (up-regulation associated cytotoxicity) on some cell types, like mesenchymal cells or endothelial cells. Considering such extensive alterations, specific expression of *BRMS1* in metastatic cells is required (27). We found that re-expression of *BRMS1* in the context of an expeditiously designed gene delivery vehicle may decline the ability of migration and invasion of metastatic adeno-carcinoma cells. This effect could, in turn, be due to the *BRMS1* function as a cellular invasion and migration inhibitory molecule. Stably *BRMS1*-transfected MDA-MB231 cell line had previously been shown to form significantly fewer metastases in all tested organs. Upon direct injection into the vasculature, fewer *BRMS1*-expressing cells attained to lungs or bone compared to the non-expressing *BRMS1* MDA-MB231 cells (17, 28). qRT-PCR analysis revealed that transfected MDA-MB231 expressed higher level of *BRMS1* compared to untreated MDA-MB231 cells. As a result, these metastatic cells have much less migratory and invasive behavior in comparison with parental cells. In concordance with the previous studies, our results revealed that *BRMS1* could significantly prevent *in vitro* migration and invasion of the human breast carcinoma cell lines (29). These unique properties of *BRMS1* gene have convinced us to employ it as an exogenous gene to prevent the invasive behavior of metastatic breast cancer cell lines. Although *BRMS1* gene could exert its anti-metastatic effects within the target cells, designing a gene delivery construct capable of cell-specific expression of this gene remains obscure.

Expression levels of *miR-200* family were evaluated in the non-metastatic and metastatic breast cancer cell lines, to unveil their expression variation in the context of the cells with metastatic behavior. Similar to the research accomplished by Burk et al. (30), we demonstrated remarkable decrease of expressing *miR-200* family members in metastatic cancer cells compared to non-metastatic cells (31), while expression of *ZEB1* and *ZEB2* genes were increased. *miR-200* family members are among the critical regulators of EMT signified by decreased expressions in metastatic cells. They target gene expression of the transcriptional repressor of *E-cadherin* (ZEB factors) and prevent their expressions. Since *ZEB1* and *ZEB2* possess *miR-200* family binding sites, the latter recognizes their binding sites in 3ˊUTR of *ZEB1* and *ZEB2* mRNA and in turn degrades mRNA molecules or prevents their translations. Our results confirmed that low levels of *miR-200* family expression lead to high levels of ZEB expression. These observations could be construed as the presence of a feedback loop between ZEB and *miR-200* family members (32). However, it should be underscored that expression level of *miR-200a* is higher than the other microRNA family members in the metastatic cell line. In agreement with the previous reports, we indicated that expression of *ZEB2* in MDA-MB231 is less increased compared to *ZEB1*. It could be rooted in the fact that *ZEB2* is the functional downstream target of *miR-200a* and higher expression of *miR-200a* caused lower expression of *ZEB2* gene (33, 34). The observed differential expression profiles of *miR-200b, miR-200c, miR-141* and *miR-429* brings about the possibility of using their MREs to restrict the expression of exogenous genes (like *BRMS1*) within the metastatic breast cancer cells and its expression in healthy tissue-derived cells. Therefore, including the MREs of these microRNAs at 3ˊUTR of an anti-metastatic gene would lead to cell-specific expression of the target gene within the metastatic breast cancer cell lines.

To confer cell type-specific expression of *BRMS1* gene under regulation of *miR-200* family MREs, designing a novel gene delivery construct seems to be vitally important. The saturation effect, spatial hindrance and in appropriate distance between MREs are among the challenges ahead of building efficient MRE regulated gene therapy constructs. In order to circumvent these snags, we used a portion of *ZEB1* 3ˊUTR which did not harbor any MRE for *miR-200a*. The performed luciferase assays revealed that MREs of *miR-200b, miR-200c, miR-141* and *miR-429* are capable to suppress expression of accompanying exogenous genes in non-metastatic breast cells without significantly compromising their expressions in the metastatic breast cancer cells. These outcomes verify the efficiency of selected *ZEB1* 3ˊUTR region to designa MRE regulated expression construct. 

This fact suggests that these MREs could be amenable regulators for therapeutic targeting of metastatic breast cells to express *BRMS1*. Our results confirmed the results of other research groups who investigated the MRE-based strategy of gene therapy for several types of malignancies including osteosarcoma (35), bladder cancer (36), uveal melanoma (37), lung (38) and prostate cancers (39). Their results suggested the possibility and effectiveness of using MREs that were down-regulated in cancer cells. It should also be pointed out that we used CMV promoter to construct the gene delivery plasmid. Potency of the cancer-specific promoters (which is used in transcriptional targeting) for driving expression of the exogenous gene is much lower than the CMV promoter. This would lead to the ineffective therapeutic influences of these vectors. Thus, using CMV promoter (potent viral promoter) along with MREs (using post-transcriptional regulation strategy for selective expression) in 3ˊUTR of the therapeutic gene could simultaneously confer potency and selectivity (38). 

## Conclusion

It could be proposed that an efficiently designed gene delivery plasmid containing both MREs and *BRMS1* gene could be a hopeful option for gene therapy against metastatic breast cancer and worthy to perform further clinical trials for metastatic cancer therapy. Such construct could provide us with the cell-specific expression of desired exogenous genes, which in turn could minimize the accompanying side-effects of the intended gene therapy.
